# The Conserved and Specific Roles of the *LUX ARRHYTHMO* in Circadian Clock and Nodulation

**DOI:** 10.3390/ijms23073473

**Published:** 2022-03-23

**Authors:** Yiming Kong, Yuxue Zhang, Xiu Liu, Zhe Meng, Xiaolin Yu, Chuanen Zhou, Lu Han

**Affiliations:** 1 The Key Laboratory of Plant Development and Environmental Adaptation Biology, Ministry of Education, School of Life Science, Shandong University, Qingdao 266237, China; ymkong@sdnu.edu.cn (Y.K.); 201912316@mail.sdu.edu.cn (Y.Z.); 201912284@mail.sdu.edu.cn (X.L.); yuxiaolin@sdu.edu.cn (X.Y.); czhou@sdu.edu.cn (C.Z.); 2 Shandong Provincial Key Laboratory of Plant Stress, College of Life Science, Shandong Normal University, Ji’nan 250300, China; zmeng@sdnu.edu.cn

**Keywords:** legume, *Medicago truncatula*, *MtLUX*, *MtFTa1*, circadian clock, photoperiodic flowering, nodulation, nyctinastic leaf movement, *Tnt1*-tagged mutant

## Abstract

*LUX ARRHYTHMO* (*LUX*) plays a key role in circadian rhythms and flowering. Here, we identified the *MtLUX* gene which is the putative ortholog of *LUX* in *Medicago truncatula*. The roles of *MtLUX*, in both the nodulation belowground and leaf movement aboveground, were investigated by characterizing a loss-of-function *mtlux* mutant. *MtLUX* was required for the control of flowering time under both long-day and short-day conditions. Further investigations showed that the early flowering in the *mtlux* mutant was correlated with the elevated expression level of the *MtFTa1* gene but in a *CO*-*like* independent manner. *MtLUX* played a conserved role in the regulatory interactions with *MtLHY*, *MtTOC1*, and *MtPRR* genes, which is similar to those in other species. Meanwhile, the unexpected functions of *MtLUX* were revealed in nodule formation and nyctinastic leaf movement, probably through the indirect regulation in *MtLHY*. Its participation in nodulation is of interest in the context of functional conservation and the neo-functionalization of the products of *LUX* orthologs.

## 1. Introduction

The circadian clock is regulated by a series of genetic programs to synchronize internal biological processes with the diurnal cycle with a period of approximately 24 h [1,2]. Our understanding of the plant clock is mostly based on the studies in Arabidopsis, which comprises hierarchical multiple feedback loops [3]. The morning-expressed clock genes contain two homologous MYB transcription factors, *CIRCADIAN CLOCK*-*ASSOCIATED 1* (*CCA1*), and *LATE ELONGATED HYPOCOTYL* (*LHY*), which negatively regulate the evening-expressed genes such as *TIMING OF CAB EXPRESSION 1* (*TOC1*), *PSEUDO-RESPONSE REGULATOR 5* (*PRR5*), *GIGANTEA* (*GI*), *EARLY FLOWERING 3* (*ELF3*), *EARLY FLOWERING 4* (*ELF4*), and *LUX ARRHYTHM*O (*LUX*) [4,5,6].

The LUX, a nuclear localized MYB domain transcription factor [7,8,9], together with ELF3, 4 comprise the evening complex (EC) [7,10]. The EC regulates many key nodes in controlling plant physiology and development, for example, hypocotyl elongation, flowering, leaf senescence, and cold response [11]. Among the core EC components, only LUX has direct DNA binding activity to repress the circadian gene expression via recruitment of the complex to the conserved LUX binding site (LBS) in promoters of targets, such as *GI*, *PRR7*, *PRR9,* and *LUX* itself [9,12,13]. The Arabidopsis *lux* mutant exhibits early flowering in both LDs and SDs, and LUX is required for normal rhythmicity of several circadian outputs in both constant light and dark conditions [7,8]. Several studies have identified and characterized the *LUX* homologs in crop species. In barley, the *eam10* mutant, also called *Hvlux1*, causes circadian defects and accelerated flowering with the up-regulation of *HvFT1* under both LDs and SDs [14]. In the garden pea, the *sn-4* mutant also confers early flowering in LDs and SDs, which is associated with the elevated expression of *FT* genes in the leaf and shoot apex [15]. In wheat, the *earliness per se 3*/*eps-3A* locus functions in both the circadian clock and photoperiodic flowering control [16,17]. In soybeans, there are three homologues of *LUX*, *GmLUXa*, *GmLUXb,* and *GmLUXc*, in which *GmLUXb* and *GmLUXc* redundantly play a role in controlling the flowering time [18].

The timing of the transition to flowering is a critical agronomic trait as its major effects on the vegetative and reproductive growth phases in plants ultimately determines crop adaptation and yield [19,20]. Plants have evolved sophisticated flowering regulatory networks that integrate environmental and endogenous cues to control when they flower [21,22,23]. One of the primary determinants is its ability to response to the photoperiod (daylength) which synchronizes the flowering time with seasonal changes [24,25,26,27]. So far, the molecular mechanism underlying plant response to the photoperiod has been extensively studied in the model plant Arabidopsis. CONSTANS (CO), a B-box zinc finger transcription factor [27], plays a central role in photoperiodic flowering by directly activating the expression of the floral integrator *FLOWERING LOCUS T* (*FT*) [28,29,30,31]. In inductive long-day (LD) conditions, the FT florigen moves via the phloem to the shoot apex, whereupon it rapidly interacts with the bZIP transcription factor FLOWERING D (FD), then induces flowering by activating the MADS box genes *SUPPRESSOR OF OVEREXPRESSION OF CONSTANS 1* (*SOC1*) and floral meristem identity (FMI) gene *APETALA 1* (*AP1*) [30,32,33]. Beyond Arabidopsis, the CO-FT pathway, which integrates the clock and light signal to regulate flowering, appears to be widely conserved. In rice, a short-day (SD) crop, the *CO*-like gene *Heading date1* (*Hd1*) is also implicated in the photoperiod measurement and photoperiod-specific regulation of rice *FT*, *Heading date 3a* (*Hd3a*) [34]. Unlike Arabidopsis *CO*, *Hd1* serves as a bifunctional regulator for activating *Hd3a* in SDs and inhibiting it in LDs [35,36]. In potato (*Solanum tuberosum*) and sugar beet (*Beta vulgaris*), *CO*-like genes also function in photoperiod responses [37,38].

Molecular genetic studies have shown the intimate linkage between photoperiodic flowering and the clock. In Arabidopsis, the altered function of circadian rhythm genes causes a reduction in photoperiodic sensitivity [39,40,41,42]. Many photoperiodic flowering genes, such as *GI*, *FLAVIN-BINDING*, *KELCH REPEAT*, *F-BOX 1* (*FKF1*), *CO,* and *CYCLING DOF FACTOR 1* (*CDF1*) exhibit rhythmic expression under the control of the circadian clock [43,44,45,46,47]. The clock allows CO accumulation to occur in the afternoon under LD [43], which is repressed by CDFs in the morning [46,47,48].

In legumes, functional analyses of several flowering time genes have demonstrated the different regulatory mechanisms. A striking difference is an absence of *FLOWERING LOCUS C* (*FLC*) clade, the floral repressors, in temperate Fabaceae family species such as *M. truncatula* and *Pisum sativum* L. (garden pea) [49,50]. In addition, the central role of the *CO*-mediated photoperiodic switch is also not conserved. In soybean, *GmCOL1a* and *GmCOL1b* are reported to be flowering repressors under LDs [51]. In addition, none of the *Medicago CO*-*lik*e (*COL*) genes are able to promote flowering or complement the Arabidopsis *co* null mutant when they are overexpressed. The knock-out of any of the *MtCOLs* does not exhibit the flowering phenotype under LDs [52]. The *FT*-like genes have been characterized in several legumes including soybean, pea, and *M. truncatula* [53,54,55]. Among the three LD-induced *MtFT* genes, *MtFTa1* is a potent floral inducer whose transcription is rapidly elevated in leaves in response to LDs and vernalization. It has been shown that flowering is late in the *fta1-1* mutant, particularly in LD conditions. However, none of the *MtFTs* possess the same diurnal expression pattern as *FT* in Arabidopsis [53], suggesting a different regulatory mechanism.

In the model legume *M. truncatula*, few clock genes have been reported and the regulation mechanism of the circadian clock remains unclear. Here, we characterized the *Tnt1* retrotransposon-tagged *mtlux* mutant in *M. truncatula*. We demonstrated that *MtLUX* plays an important role in maintaining clock gene expression and controlling photoperiodic flowering through the up-regulation of *MtFTa1*. In addition, *MtLUX* was involved in the nodule formation and nyctinastic leaf movement, suggesting its specific function in the legume species.

## 2. Results

### 2.1. Identification and Phenotypic Characterization of the Early Flowering Mutants in M. truncatula

**Figure 1:**
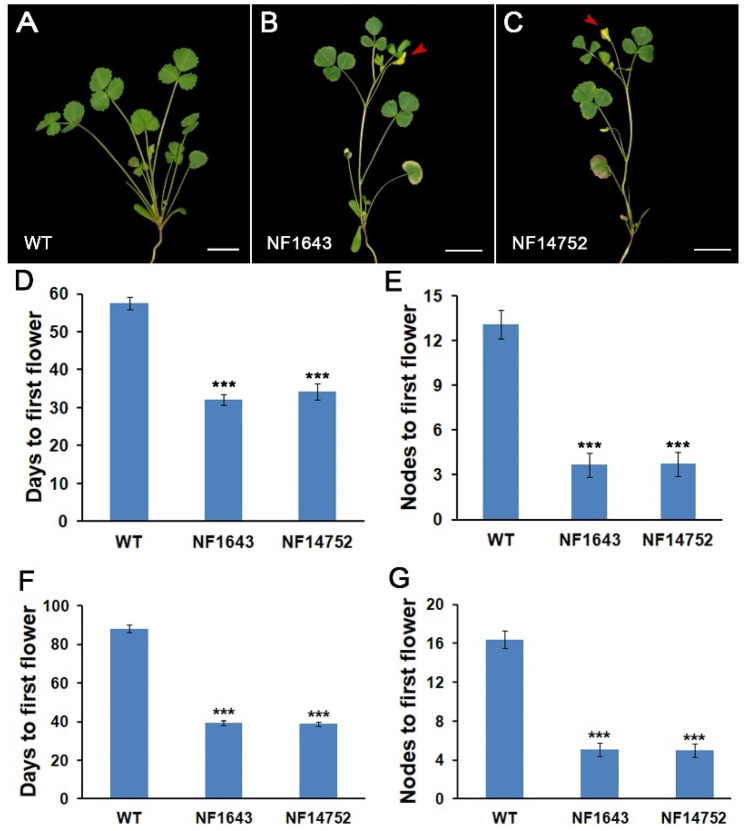
Early flowering phenotype in the mutant plants. (**A**–**C**) 5-week-old plants of the wild-type (WT) (**A**) and mutants (**B**,**C**) in LDs. Arrows indicate the flowers in the mutants. Bars = 1 cm. (**D**) The days to the first flower of WT and mutants in LDs. (**E**) Nodes to the first flower of WT and mutants in LDs. (**F**) Flowering time in days to the first flower of WT and mutant plants in SDs. (**G**) Nodes to the first flower of WT and mutant plants in SDs. The flowering time in (**D**–**G**) is shown as means ± SD (*n* = 10). ***: means differ significantly (*p* < 0.001).

To study the regulation mechanism of flowering in M. truncatula, a Tnt1 retrotransposon-tagged mutant population was screened [56]. Two independent mutant lines, NF1643 and NF14752, with the early flowering phenotype were identified (Figure 1A–C). Under long-day conditions (LDs), the flowering time of mutants (32.1 ± 1.4 d of NF1643 and 34.2 ± 2.0 d of NF14752 after seed germination) was advanced by 3–4 weeks, compared to that of the wild-type (57.6 ± 1.65 d after seed germination) (Figure 1D). The first flower was observed on the 3rd or 4th node on the primary shoot axis in the mutants but appeared on the 13th node in the wild-type (Figure 1E). In addition, the mutants also flowered significantly earlier than the wild-type plants in short-day conditions (SDs) (Figure 1F,G), indicating that flowering time in the mutants was accelerated under both LDs and SDs.

### 2.2. Molecular Cloning of MtLUX

**Figure 2:**
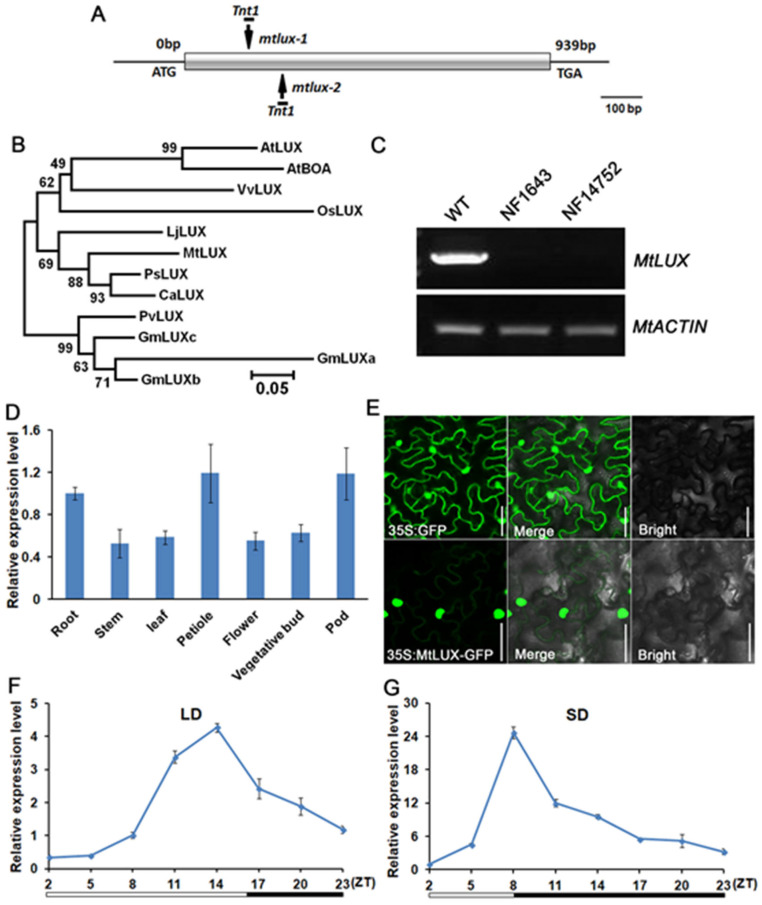
Molecular cloning and expression pattern of *MtLUX* in *M. truncatula*. (**A**) Schematic representation of the *MtLUX* gene structure and *Tnt1* insertion site. Box represents exon and lines represent 5′ and 3′-UTRs. (**B**) A neighbor-joining tree of MtLUX and LUX orthologs from other species including *A. thaliana* (AtLUX and AtBOA), *Oryza sativa* (OsLUX), *Vitis vinifera* (VvLUX), *Cicer arietinum* (CaLUX), *Phaseolus vulgaris* (PvLUX), *Pisum sativum* (PsLUX), *Lotus japonicus* (LjLUX), and *Glycine max* (GmLUXa, b and c). (**C**) RT-PCR analysis of full-length transcript of *MtLUX* in the WT and *mtlux* mutants. Actin was used as the control. (**D**) The expression level of *MtLUX* in different organs. *MtUBIQUITIN* was used as the internal control. Values are shown as means ± SD of three biological replicates. (**E**) The subcellular localization of MtLUX-GFP fusion proteins. Free GFP as the control. Bars = 50 um. (**F**,**G**) *MtLUX* transcript levels in long-day (16 h light/8 h dark) (**F**) and short-day (8 h light/16 h dark) conditions (**G**) in 4-week-old WT plant. *MtUBIQUITIN* was used as the internal control. Values are shown as means ± SD of three biological replicates. White and black bars at the bottom indicate periods of light and dark, respectively.

To identify the gene responsible for the mutant phenotype, thermal asymmetric interlaced-PCR (TAIL-PCR) was performed to recover the flanking sequence tags (FSTs) of *Tnt1* retrotransposon in the mutants. Based on the results of PCR genotyping, one flanking sequence was confirmed to segregate both mutants. A full-length genomic sequence of 939 nucleotides was obtained using this FST to search against the *M. truncatula* genomic sequences in the NCBI database. By reverse transcription (RT)-PCR and sequence analysis, a full-length coding sequence (CDS) of 939 nucleotides was obtained, which revealed that the gene, *Medtr4g064730*, is intronless (Figure 2A). Further analysis showed that a *Tnt1* retrotransposon was inserted in the exon and the full-length transcript was absent in mutant alleles (Figure 2A,C). Bioinformatic analysis showed that *Medtr4g064730* encoded a MYB domain protein and was the putative ortholog of the Arabidopsis *LUX* gene (Figure S1). Therefore, this gene was named *MtLUX*. Phylogenetic analysis revealed that *MtLUX* is evolutionarily closer to the *LUX* orthologs in the legume species, such as *Cicer arietinum*, *Pisum sativum,* and *Lotus japonicus* (Figure 2B). Protein alignment revealed highly conserved amino acid sequences, especially on the DNA-binding myb-like domain flanked by a short, moderately conserved N-terminal domain (Figure S2).

### 2.3. MtLUX Expression Pattern and Subcellular Localization

To explore the expression pattern of *MtLUX*, we examined the transcriptional level of *MtLUX* in various organs via qRT-PCR. The result showed that *MtLUX* was highly expressed in root, petiole, and pod (Figure 2D). To study the cellular localization of MtLUX, we fused the C-terminus of the MtLUX protein with green fluorescent protein (GFP) under the control of the *Cauliflower mosaic virus* (CaMV) 35S promoter and transformed into tobacco (*Nicotiana benthamiana*) epidermal cells by the *Agrobacterium* infiltration method. Using fluorescence microscopy, we observed the 35S:GFP was localized in both the cytoplasm and nucleus of epidermal cells, while the 35S:MtLUX-GFP fusion protein was localized primarily in the nucleus, indicating that MtLUX probably plays a role in nuclear in the *M. truncatula* (Figure 2E).

To investigate the diurnal expression of *MtLUX*, the leaves of WT were collected at 3 h intervals from ZT2 (ZT: Zeitgeber time) for qRT-PCR analysis. Under both long-day and short-day entrainment, the accumulation of *MtLUX* mRNA exhibited strong rhythmic expression with a peak early at night and a trough at dawn (Figure 2F,G), indicating that *MtLUX* is an evening component under diurnal control. The diurnal expression pattern of *MtLUX* implied that the endogenous circadian clock might regulate the transcriptional level of *MtLUX*. To confirm this hypothesis, we examined the expression pattern of *MtLUX* in the plants grown in constant light conditions. The result showed that a clear circadian rhythm of *MtLUX* with the peak time appeared during the subjective night (Figure S3).

### 2.4. MtLUX Is Negatively Correlated with Transcript Levels of MtFTa1 Gene but Not MtCOL Genes

**Figure 3:**
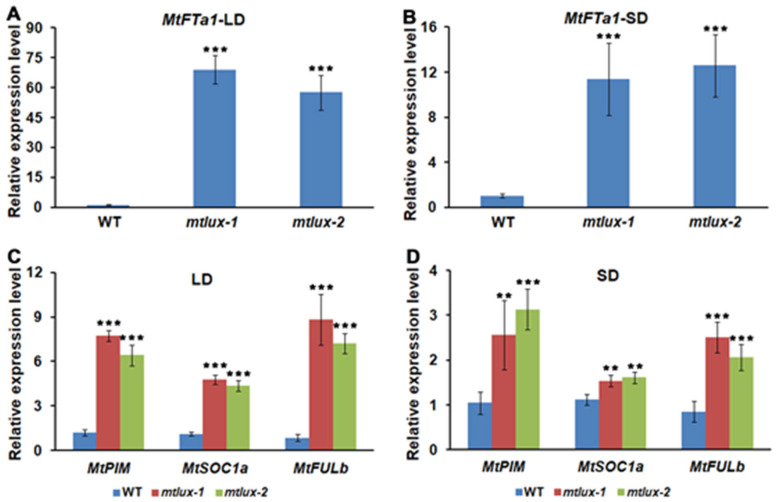
Transcript levels of flowering control genes. (**A**,**B**) Expression level of *MtFTa1* in long-day (**A**) and short-day (**B**) in the leaves of 4-week-old wild-type and *mtlux* mutant. (**C**,D) Expression level of *MtPIM*, *MtSOC1a*, and *MtFULb* in long-day (**C**) and short-day (**D**) in the leaves of 4-week-old wild-type and *mtlux* mutant. Values are shown as means ± SD of three biological replicates. **, ***: means differ significantly (*p <* 0.01, *p <* 0.001).

To investigate the mechanism of the early-flowering phenotype observed in the *mtlux* mutant (Figure 1), the expression level of *MtFT* was analyzed. Among the five characterized *FT-like* genes in *M. truncatula*, *MtFTa1* is a key regulator of flowering time [53]. The abundance of the *MtFTa1* transcript was markedly elevated in the leaves of *mtlux*, compared to that in the wild-type in LDs, SDs, and constant light conditions (Figure 3A,B and Figure S4A). This result suggested that the early flowering defects in *mtlux* may be induced by the ectopic expression of *MtFTa1* under different growth conditions. Furthermore, the transcript level of several flowering control genes was examined. *MtSOC1a* and *MtFULb* were selected because that they are the target genes of *MtFTa1,* and can be induced in *MtFTa1* over-expressing plant [57]. Additionally, the marker gene of the floral transition, *MtPIM*, was also chosen for analysis. The results showed that the transcriptional abundance of three genes was significantly increased in the leaves of the *mtlux* mutant (Figure 3C,D), which further confirmed the role of *MtFTa1* in the early flowering phenotype of mutants.

In addition, three orthologs of Arabidopsis *CO*, *MtCOLa*, *MtCOLb,* and *MtCOLc*, were identified in *M. truncatula*. The qRT-PCR data showed that the transcript of *MtCOLa*, *b* was decreased in LD and *MtCOLa* was decreased in SD (Figure S5). Under LL, we found markedly decreased and defected rhythmic expression of *MtCOLa* (highest sequence similarity to *CO* in Arabidopsis) in the *mtlux* mutant. However, the *MtCOLa* exhibited a clearly rhythmic expression pattern with a peak at subjective dawn in the wild-type (Figure S4B). In a previous study, *MtCOL* genes do not participate in the induction of photoperiodic flowering in *M. truncatula* [52]. Therefore, the promotion of flowering in *mtlux* is associated with the induction of the *MtFTa1* instead of *MtCOL*.

### 2.5. The Expression Pattern of Genes Associated with Circadian Clock Is Altered in Mtlux Mutants

**Figure 4:**
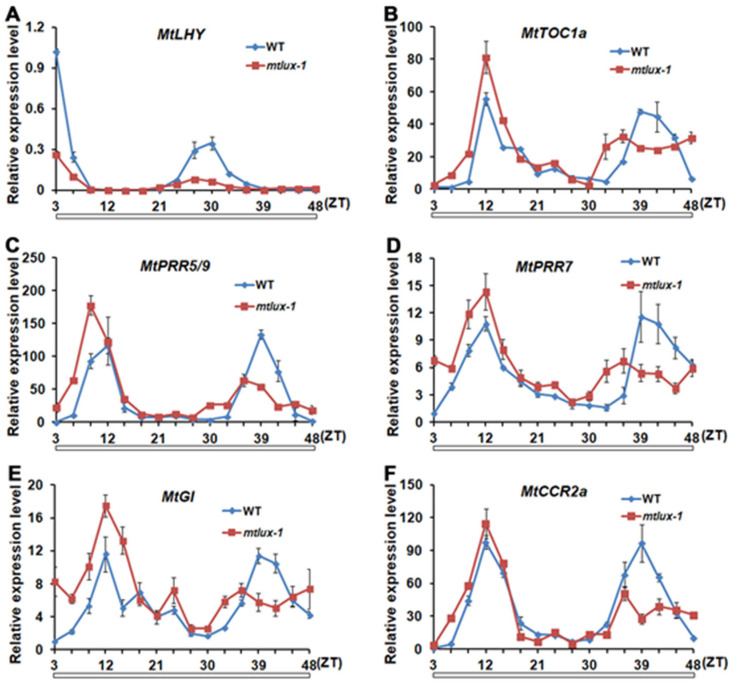
Regulation of *MtLUX* on circadian expression of clock genes in continuous light. The transcriptional behaviors of *MtLHY* (**A**), *MtTOC1a* (**B**), *MtPRR5/9* (**C**), *MtPRR7* (**D**), *MtGI* (**E**), and *MtCCR2a* (**F**) in the leaves of 4-week-old WT and *mtlux-1* plants grown under constant light conditions. Values are shown as means ± SD of three biological replicates. White bars at the bottom indicate periods of constant light.

The expression pattern of *MtLUX* exhibited a diurnal and circadian rhythm, implying that *MtLUX* may play a role in maintaining normal clock rhythmicity. To confirm this hypothesis, the rhythmic expressions of several clock genes including *MtLHY*, *MtTOC1a*, *MtPRR5/9*, *MtPRR7*, *MtGI*, and *MtCCR2a* were analyzed in the wild-type and *mtlux-1* mutant under constant light conditions for 2 d (Figure 4). The results showed that the circadian clock genes maintained the robust rhythmic cycles in the *mtlux-1* mutant in the first day. However, on the second day, the waveforms were rough, and the amplitudes were reduced with an obvious phase advance of approximately 3 h in the *mtlux-1* mutant, displaying a period shortening effect. These observations indicated that the expression of circadian clock genes is severely compromised in the *mtlux* mutant under constant light conditions, suggesting the potential role of *MtLUX* in controlling the clock rhythmicity. Moreover, the abundance of *MtLHY* was significantly decreased throughout the daytime, particularly at the peak time (Figure 4A), and the repression of *MtLHY* is also consistent with the short period phenotype of the *mtlux-1* mutant. Conversely, the expression levels of *MtTOC1a*, *MtPRR5/9*, *MtPRR7*, *MtGI,* and *MtCCR2a* were higher in the *mtlux-1* mutant than those in the wild-type at the most time points during the first day (Figure 4B–F), implying the different regulatory roles of *MtLUX* in circadian components.

### 2.6. The Transcriptomic Profiles of the Loss-of-Function of MtLUX

To better understand the function of *MtLUX* in *M. truncatula*, we performed RNA sequencing (RNA-seq) using 4-week-old wild-type and *mtlux-1* plants. Three biological replicates of leaves were harvested at zeitgeber time (ZT) 15, which is the peak expression time of *MtLUX* in long-day conditions. Each replicate generated approximately 19–20 million raw reads and they were filtered and aligned against the annotated genome for gene quantification. The differentially expressed genes (DEGs) were identified by more than 2-fold expression alterations and less than 0.001 of false discovery rate (FDR). In total, 1580 DEGs were found in *mtlux-1* vs. WT transcriptomes, among which 421 were up-regulated and 1159 were down-regulated in the *mtlux-1* mutant (Figure S6). Functional assignment of the DEGs by Gene Ontology (GO) analysis revealed that widely enriched terms comprised various regulatory networks (Figure S7).

Of particular interest are the 26 DEGs that are involved in the circadian rhythm pathway, which includes many key clock-associated genes listed in Table 1. The expression levels of multiple members of the *PRR* family, including *MtTOC1a*, *MtTOC1b*, *MtPRR5/9*, *MtPRR3*, and *MtELF3*, *MtGI*, *MtCCR2*, were elevated. Additionally, the *MtLHY* expression was decreased in the *mtlux-1* mutant, which is consistent with qRT-PCR data. Notably, many DEGs correlated with clock-regulated outputs were present in the transcriptome, such as encoding products associated with photosynthesis-antenna proteins and photosynthesis, carbon fixations, starch and sucrose metabolism, amino acid metabolism and nitrogen metabolism, and nitrate transporter (Tables S2–S6). Taken together, the results of transcriptomic profiling analysis suggest that *MtLUX* might regulate the clock and its outputs by transcriptional regulation.

### 2.7. MtLUX Indirectly Regulates Nodulation and Nyctinastic Leaf Movement

According to the *Medicago truncatula* Gene Expression Atlas (MtGEA), the expression of *MtLUX* was highly induced in nodules upon *Sinorhizobium meliloti* 1021 inoculation (Figure S8). The transcript level of *MtLUX* was increased approximately 20-fold in nodules at 10 days post inoculation (dpi), then decreased at 14 dpi, implying that *MtLUX* may be involved in the nodulation process in *M. truncatula*.

**Figure 5:**
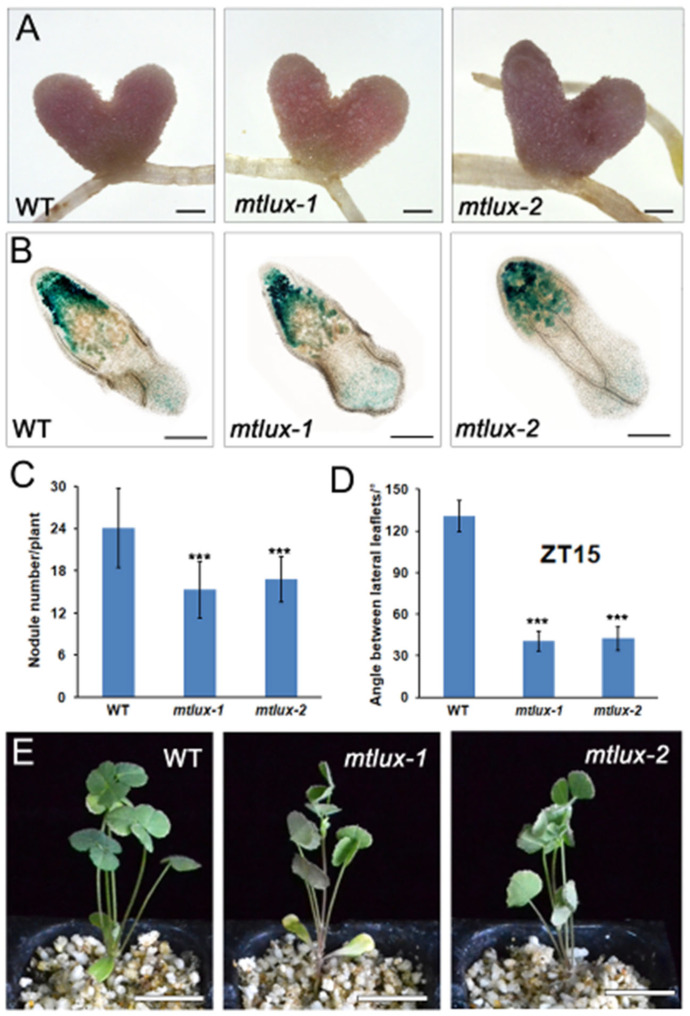
*MtLUX* gene is involved in nodulation and nyctinastic leaf movement during symbiosis. (**A**) The 21 dpi nodules in wild-type and *mtlux* plants. Bars = 1 mm. (**B**) The nodules in wild-type and *mtlux* plants were sliced into sections for β-galactosidase staining. Bars = 1 mm. (**C**) Nodule number in wild-type and *mtlux* plants counted 21 days after inoculation. Values shown as means ± SD (*n* = 20). ***: means differ significantly (*p <* 0.001). (**D**) The angle was measured between two lateral leaflets of the first fully expanded trifoliate in 21 days wild-type and *mtlux* seedlings at ZT15 after inoculation. Values are shown as means ± SD (*n* = 15). (**E**) At ZT15, the leaf movement of 21 days wild-type and *mtlux* plants after inoculation. Bar = 2 cm.

To characterize the potential role of *MtLUX* during symbiosis, wild-type and *mtlux* mutant plants were inoculated with rhizobia bacteria *S. meliloti* 1021 harboring a hemA:lacZ reporter gene and grown under long-day conditions for 3 weeks. The shape and size of nodules in *mtlux* mutants exhibited no obvious defects after 3 weeks of inoculation, compared with those in the wild-type (Figure 5A). The X-gal staining of lacZ-expressing rhizobia revealed that nodules of the mutant harbored similar rhizobia to those of the wild-type (Figure 5B). However, the number of nodules was significantly reduced in the *mtlux* mutants (Figure 5C). *M. truncatula* leaflets displayed nyctinastic movement: they opened during the day and closed in the night. The leaf movement did not show the obvious difference between the un-inoculated plants of the wild-type and *mtlux* mutant. It is interesting to note that the leaflets of the mutant started to close earlier under symbiotic conditions. At ZT15, the angle between two lateral leaflets in the *mtlux* mutant plants reached approximately 40°, while the angle in the wild-type remained at 130° (Figure 5D,E). This observation indicates that both nodulation and leaf nyctinastic movement is affected in the *mtlux* mutant plants.

## 3. Discussion

The circadian clock is a self-sustaining oscillator resulting from an intricate network of interlocked transcriptional and translational feedback loops to enhance the adaptation and survival of plants [19,20]. The studies of the circadian clock in plants mainly focused on the model species such as *A.thaliana*. However, the regulatory mechanism of the circadian clock in the legume species is largely unknown. In this study, we identified *MtLUX* as the ortholog of Arabidopsis *LUX* in the legume model species *M. truncatula*. Our study clearly showed that *MtLUX* transcripts were entrained by light/dark cycles and constant light conditions to display diurnal and circadian rhythms (Figure 2 and Figure S3). Consistently, two evening elements (AAAATATCT) were found in the promoter of *MtLUX* at 445 bp and 194 bp upstream of the translational start site ATG. *MtLUX* showed obvious rhythmic expression under light/dark cycles. After being released into constant light conditions, *MtLUX* transcripts was gradually decreased, indicating damped expression rhythms in LL, as seen in that of the *GmLUXc* gene in soybeans [18]. Interestingly, *MtLUX* had a LUX binding site (LBS, GATWCG) in the promoter region of 205 bp upstream of ATG, like *LUX* and *GmLUXc*, whose LBS is located in the promoter of 545 bp and 321 bp upstream of ATG, respectively. Such self-regulation of its expression by a negative feedback mechanism may explain the damped expression of *MtLUX* after being transferred to LL.

In Arabidopsis, *CO* plays a central role in photoperiod measurement. It integrates the circadian clock and light signal to induce *FT* expression, and then to promote flowering. However, the regulation of flowering in *M. truncatula* appears to act in a *CO*-*like* independent manner. Firstly, recent research revealed that three *Medicago CO*-*like* genes, *MtCOLa*, *MtCOLb,* and *MtCOLc*, do not participate in the induction of photoperiodic flowering [52]. Secondly, the expression level of *MtFTa1* is significantly reduced, while several *MtCOL* genes are unaffected in *MtCDFd* overexpressing transgenic plants which exhibit delayed flowering [58]. In this study, loss-of-function of *mtlux* mutants displayed accelerated flowering and the shortened vegetative phase under both LD and SD (Figure 1). However, the transcript levels of three *MtCOL* genes were not induced in the *mtlux* mutant (Figures S4B and S5), although strong activation of the *MtFTa1* gene was detected either in the light/dark cycles or constant light conditions (Figure 3A,B and Figure S4A). These results are consistent with those in the pea, in which it also lacks the functional *COL* genes in photoperiod responsive flowering. Hence, how the clock and light signal are integrated for *FT* regulation in *M. truncatula* and other legumes remains to be investigated.

The clock components were classified according to their phases of expression. In Arabidopsis, the evening-phased component contains three proteins, LUX, ELF3, and ELF4, which form the so-called “evening complex” (EC). Among the EC, LUX functions as a repressor by binding directly to targets [9,59]. In the *mtlux* mutant, circadian expression patterns of multiple clock-associated genes dampened under constant light conditions (Figure 4). These findings indicate that the circadian rhythmicity in *mtlux* is compromised, suggesting the important roles of *MtLUX* in the maintenance of circadian rhythms in *M. truncatula*. This observation is accompanied by the early flowering phenotype irrespective of day length, which is similar to that in Arabidopsis, pea, and barley [7,14,15]. In the *mtlux* mutant, the expression of *MtLHY*/*MtCCA1* is down-regulated and *MtPRR5/9*, *MtPRR7*, *MtTOC1a*, *MtGI*, and *MtCCR2a* are up-regulated on the first day of LL (Figure 4), implying that they may not act in a linear pathway. This result is consistent with the role of *LUX* as a positive regulator of *CCA1*/*LHY* and a negative regulator of *TOC1* (*PRR1*), *PRR9*, *7* and *GI* in Arabidopsis [6,7]. These results indicate the evolutionary conservation and distinct function of *LUX* in Medicago and Arabidopsis in the circadian clock outputs regulations.

**Figure 6:**
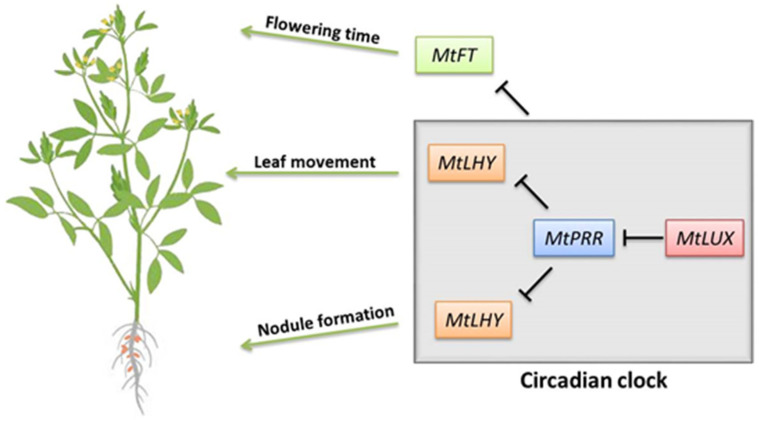
Model for the proposed role of MtLUX in *M. truncatula*. MtLUX is responsible for the down-regulation of *MtPRRs* and the up-regulation of *MtLHY*. The MtLUX-associated circadian clock is involved in the regulation of nodule formation, leaf movement, and flowering time.

Many clock oscillators have been confirmed as the key role in the domestication of agricultural crops via influencing key agricultural traits, such as flowering time, yield, resistance to stress, and so on [60]. Particularly, several studies showed that multiple clock outputs, including photosynthesis, carbohydrates, and nitrogen metabolism, display the feedback regulation to the function of the core clock itself [20,61,62,63,64,65]. In Arabidopsis, the clock oscillators function to control the N-assimilation pathway due to the direct regulation of CCA1 on N-assimilation genes, in turn, the nitrogen source such as glutamate (Glu) or Glu-derived signals act as input to the clock via the core regulator *CCA1* [61]. Recently, we reported that a loss-of-function mutant of *MtLHY* leads to a reduction in the number of nodules through its regulation on flavonoid biosynthesis, resulting in a diminished ability to assimilate nitrogen. Meanwhile, the leaf nyctinastic movement in the *mtlhy* mutant is further influenced by the availability of nitrogen produced by the nodules, indicating the communication between the nodulation process and circadian clock [66]. In this study, the decreased number of nodules and the irregulated nyctinastic leaf movement were also observed in *mtlux* mutants, which is similar to those in the *mtlhy* mutant. In addition, both the *mtlux* and *mtlhy* mutant exhibited the early flowering phenotype and multiple DEGs associated with amino acid metabolism and nitrogen metabolism [66]. Moreover, the expression of *MtLHY* was significantly reduced in *mtlux*. A plausible hypothesis, therefore, is that the defects in *mtlux* mutants were probably due to the decreased expression level of *MtLHY* as the proposed working model illustrated in Figure 6, although MtLUX probably indirectly activates the transcriptional level of *MtLHY*.

The homologs of *LUX* have been identified in several species, including garden peas, lotus, and cereals, such as barley and wheat, and they were involved in the circadian clock and photoperiod responsiveness [7,14,15]. The similar misregulation of *CCA1*/*LHY*, *TOC1*, and *PRR* genes were displayed in mutants of *LUX* orthologs among species, suggesting that LUX plays a conserved role in the regulatory interactions within the circadian clock. Meanwhile, the unexpected functions of MtLUX were revealed in the nodule formation and leaf movement, probably through the indirect regulation of *MtLHY*. These data shed new light on the conserved and specific roles of *MtLUX* in the orchestration of circadian oscillator among species and offer some insight into the impact of the circadian clock components on nodulation in the legume species.

## 4. Materials and Methods

### 4.1. Plant Materials and Growth Conditions

All experiments described in this study used *M. truncatula* ecotype R108. *mtlux1-1*, *-2* mutants were isolated from forward-screening a tobacco (*Nicotiana tabacum*) *Tnt1* retrotransposon-tagged mutant collection [56] of *M. truncatula*. For flowering time measurement, plants were grown under either long- (16 h) or short-day (8 h) photoperiods in the greenhouse maintained at 22 °C, with between 70 and 80% relative humidity, and a light intensity of approximately 150 μmol/m^2^/s. For circadian clock expression analyses of *MtLUX* and the other clock genes, the plants were grown in a light incubator with between 70 and 80% relative humidity, 22 °C, and a light intensity of 90 μmol/m^2^/s. They were grown under long-day (16 h) photoperiods for 3 weeks, then transferred to short-day (12 h) for 1 week followed by 2 days constant light conditions for sampling. For diurnal rhythmic analysis of *MtLUX*, the wild-type plants were grown under long-day (16 h) or short-day (8 h) periods for 4 weeks and then the leaves were collected for analysis.

### 4.2. Phylogenetic Analysis

Multiple sequence alignments were performed using Clustal Omega with default parameters. Then, the phylogenetic trees were constructed using the MEGA7.1 program by the Neighbor Joining (NJ) method with 1000 bootstrap replicates (http://www.megasofware.net/ accessed on 10 March 2020).

### 4.3. Subcellular Localization of MtLUX

PCR was performed to amplify *MtLUX* CDS (primers listed in Supplementary Table S1) and then cloned into the pENTR/D-TOPO vector (Invitrogen, Carlsbad, CA, USA). The insert was then recombined into the binary vector pEarleyGate 103 using the Gateway LR reaction (Invitrogen, Carlsbad, CA, USA) [67]. The destination vector was finally introduced into the disarmed *Agrobacterium tumefaciens* EHA105 strain, which then transformed into tobacco epidermal cells. The GFP protein and the MtLUX-GFP fusion protein were examined using confocal microscopy LSM 880 (Zeiss, Jena, Germany).

### 4.4. Root Nodule Induction

Wild-type and *mtlux* mutant plants were grown in a mixture of pearlite/sand (3:1 ratio) under a 16 h photoperiod at 22 °C with a relative humidity from 60–70% and a light intensity of 90 μmol/m^2^/s. The *S. meliloti* strain 1021 was incubated in TY medium supplemented with 6 mM CaCl_2_, 200 μg/mL streptomycin and 10 μg/mL tetracycline and shaken at 28 °C overnight until the OD600 value reached to 1.0. Three days after sowing, the seedlings were inoculated with 5 mL of *S. meliloti* 1021 strain suspension insufficient water of OD600 of 0.1. After 3 weeks, the nodules were counted, and leaf angles were recorded. The LacZ activity of nodules was performed as previously described [68].

### 4.5. RNA Isolation and Quantitative Realtime PCR (qRT-PCR) Analysis

The leaves and the other organs of 4-week-old plants were collected for total RNA isolation using the Trizol-RT Reagent (Invitrogen, Carlsbad, CA, USA). The quantitative and qualitative value of RNA was measured using the Nanodrop 2000 Spectrophotometer (Thermo Fisher Scientific Inc., Waltham, MA, USA). Three micrograms of total RNA were reverse transcribed into cDNA using the Roche RNA Reverse Transcription Kit (Roche Molecular Systems, Inc., Branchburg, NJ, USA). Subsequent qRT-PCR was performed on Bio-Rad CFX Connect^TM^ based on Roche SYBR-green fluorescence dye (FastStart Essential DNA Green Master) with *MtUBIQUITIN* as the reference. All the qRT-PCR primers are listed in Supplementary Table S1. For transcriptomic analysis, three biological replicates of leaves were collected at ZT15 from 4-week-old wild-type and *mtlux-1* plants after sowing in LDs. RNA samples were sequenced on a BGISEQ-500 platform at the BGI Genomics Institute (BGI, Shenzhen, China).

### 4.6. Accession Numbers

GenBank accession numbers for the genes in this article are as follows: MtLUX: Medtr4g064730; MtSOC1a: Medtr7g075870; MtFULb: Medtr4g109830; MtPIM: Medtr8g066260; MtFTa1: Medtr7g084970; MtCOLa: Medtr7g018170; MtCOLb: Medtr1g013450; MtCOLc: Medtr3g105710; MtLHY: Medtr7g118330; MtPRR5/9: Medtr3g092780; MtPRR7: Medtr1g067110; MtTOC1a: Medtr4g108880; MtGI: Medtr1g098160; MtCCR2a: Medtr4g070080; LUX: AT3G46640; AtBOA: At5G59570; PsLUX: KJ801796; GmLUXa: Glyma01g36730; GmLUXb: Glyma12g06406; GmLUXc: Glyma11g14490; LjLUX: Lj3g3v3214340; OsLUX: XP_025878289; VvLUX: XP_010650997; CaLUX: XP_004507164; PvLUX: XP_007132043.

## Figures and Tables

**Table 1:** Differentially expressed genes (DEGs) of circadian rhythm pathway in the leaves of the *mtlux-1* mutant compared to the wild-type.

Gene ID	Description	Gene Name	log2(*mtlux-1*/WT)	FDR
Medtr1g098140	chalcone and stilbene synthase family protein	-	11.62570884	2.24 × 10^−7^
Medtr7g016720	chalcone and stilbene synthase family protein	-	11.09011242	6.06 × 10^−5^
Medtr3g082630	B-box type zinc finger protein	-	3.240051088	7.12× 10^−9^
Medtr4g061360	PRR response regulator	*MtPRR3*	2.841248051	3.09 × 10^−121^
Medtr3g037390	timing of cab expression 1/PRR response regulator	*MtTOC1b*	2.182937767	2.54 × 10^−16^
Medtr8g105590	flavin-binding kelch repeat F-box protein, putative	*MtFKF1*	1.913744267	4.36 × 10^−6^
Medtr3g091340	CCT motif protein	-	1.428077186	7.93 × 10^−16^
Medtr4g108880	two-component response regulator-like APRR7 protein	*MtTOC1a*	1.397727614	9.32 × 10^−12^
Medtr1g098160	gigantea protein 1B	*MtGI*	1.326863054	2.20 × 10^−124^
Medtr3g092780	PRR response regulator	*MtPRR5/9*	1.315561529	1.15 × 10^−19^
Medtr3g103970	early flowering protein	*MtELF3*	1.212819095	4.07 × 10^−6^
Medtr7g083540	zinc finger constans-like protein	-	1.155346537	0.0002833
Medtr4g046640	B-box type zinc finger protein	-	−12.52160044	0.0001545
Medtr4g008050	B-box type zinc finger protein, putative	-	−2.731240606	1.29 × 10^−32^
Medtr7g110810	helix loop helix DNA-binding domain protein	*MtPIF4like*	−2.351726642	2.37 × 10^−12^
Medtr1g067000	myb transcription factor	-	−2.182338147	5.41 × 10^−233^
Medtr2g089310	B-box type zinc finger protein	-	−1.935687612	9.49 × 10^−58^
Medtr7g118330	late elongated hypocotyl-like protein	*MtLHY*	−1.689747281	0
Medtr5g021580	salt tolerance-like protein	-	−1.379673589	3.91 × 10^−48^
Medtr1g013450	zinc finger constans-like protein	*MtCOLb*	−1.361552093	6.45 × 10^−145^
Medtr7g018170	zinc finger constans-like protein	*MtCOLa*	−1.324077697	1.87 × 10^−102^
Medtr5g058920	phosphatidylinositol-4-phosphate 5-kinase	-	−1.156725504	6.44 × 10^−6^
Medtr1g016920	early flowering protein, putative	*MtELF3-like*	−1.063984456	4.27 × 10^−5^
Medtr3g449770	transcription factor	*MtPIF3like*	−1.060477166	2.44 × 10^−60^
Medtr1g084980	phytochrome-interacting factor 3.1	*MtPIF5like*	−1.020982564	1.78 × 10^−26^
Medtr6g053620	ubiquitin-protein ligase, putative	-	−1.411551885	0.0007789
Medtr1g067110	two-component response regulator-like APRR7 protein	*MtPRR7*	0.79438414	9.02 × 10^−39^
Medtr4g070080	RNA-binding (RRM/RBD/RNP motif) family protein	*MtCCR2a*	1.794137127	3.88 × 10^−167^
Medtr4g070190	RNA-binding (RRM/RBD/RNP motif) family protein	*MtCCR2b*	1.692850978	1.572 × 10^−32^
Medtr4g070140	RNA-binding (RRM/RBD/RNP motif) family protein	*MtCCR2c*	2.237543475	9.1 × 10^−103^

## Data Availability

The data presented in this study are available in supplementary material here.

## Supplementary Materials

The following supporting information can be downloaded at: https://www.mdpi.com/article/10.3390/ijms23073473/s1.
