# Supplementary material for: The Conserved and Specific Roles of the LUX ARRHYTHMO in Circadian Clock and Nodulation
Source: Int J Mol Sci. 2022 Mar 23;23(7):3473. doi: 10.3390/ijms23073473 (PMC8998424; doi:10.3390/ijms23073473)
Supplement: Supplementary file 1 [file ijms-23-03473-s001.zip › Supplemental figures and figure legends.pdf]

Supporting Informations

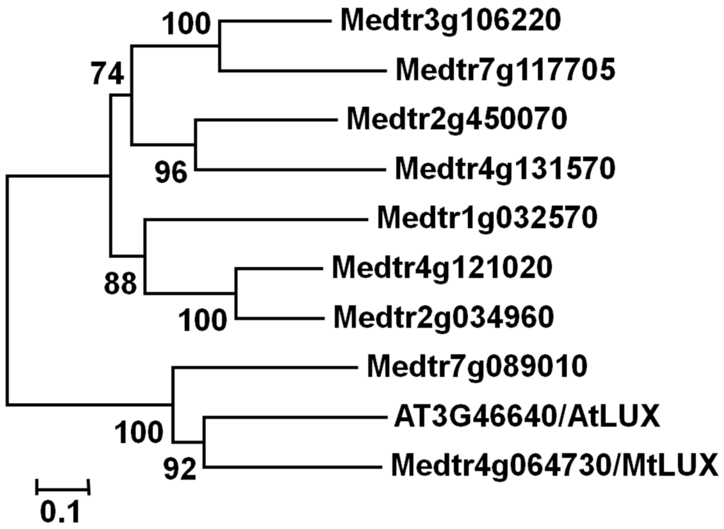

Figure S1 | Phylogenetic analysis of AtLUX protein and MtLUX like proteins from *M. truncatula.* The most strongly related *M. truncatula* protein to AtLUX is the product of *Mt4g064730* (*MtLUX*). Bootstrap value supporting each node is shown.

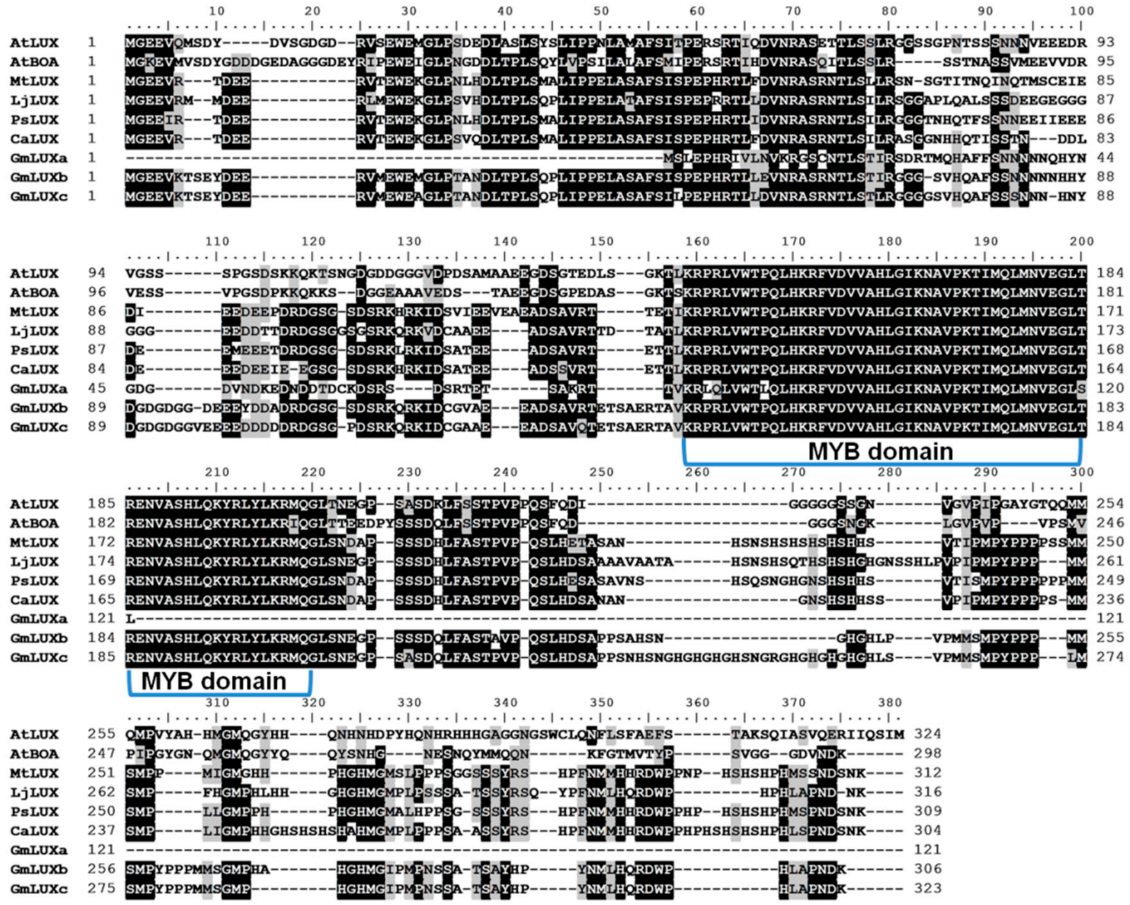

Figure S2 | Amino acids alignment for LUX-Like proteins.

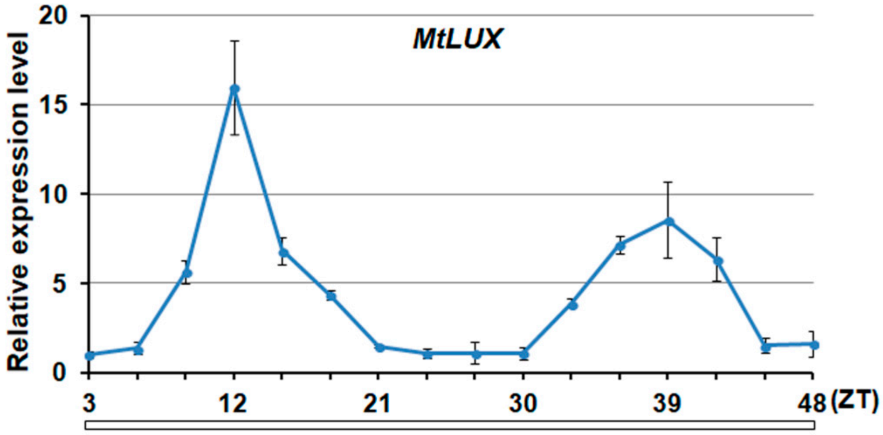

Figure S3 | The expression pattern of *MtLUX* in the leaves of 4-week-old WT under constant light condition. Values shown as means ± SD of three biological replicates. *MtUBIQUITIN* was used as the internal control. White bar at the bottom indicates period of constant light.

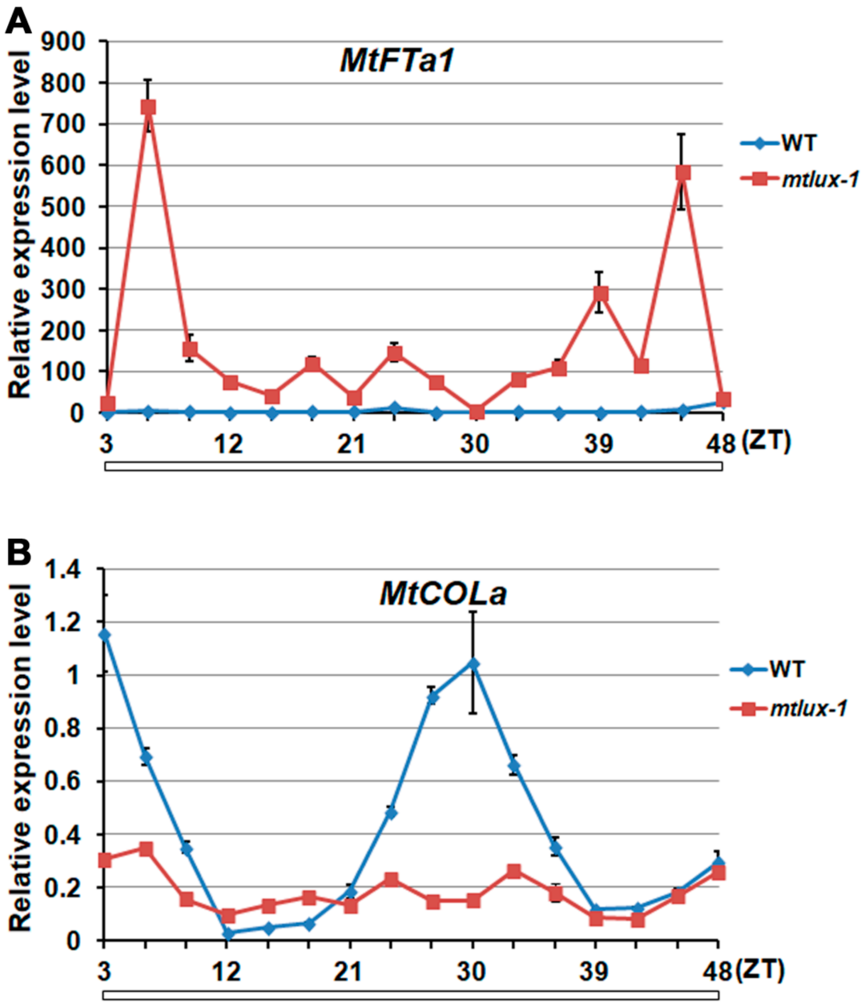

Figure S4 | The expression pattern of *MtFTa1* (A) and *MtCOLa* (B) in the leaves of 4-week-old WT under constant light condition. Values shown as means ± SD of three biological replicates. *MtUBIQUITIN* was used as the internal control. White bars at the bottom indicate periods of constant light.

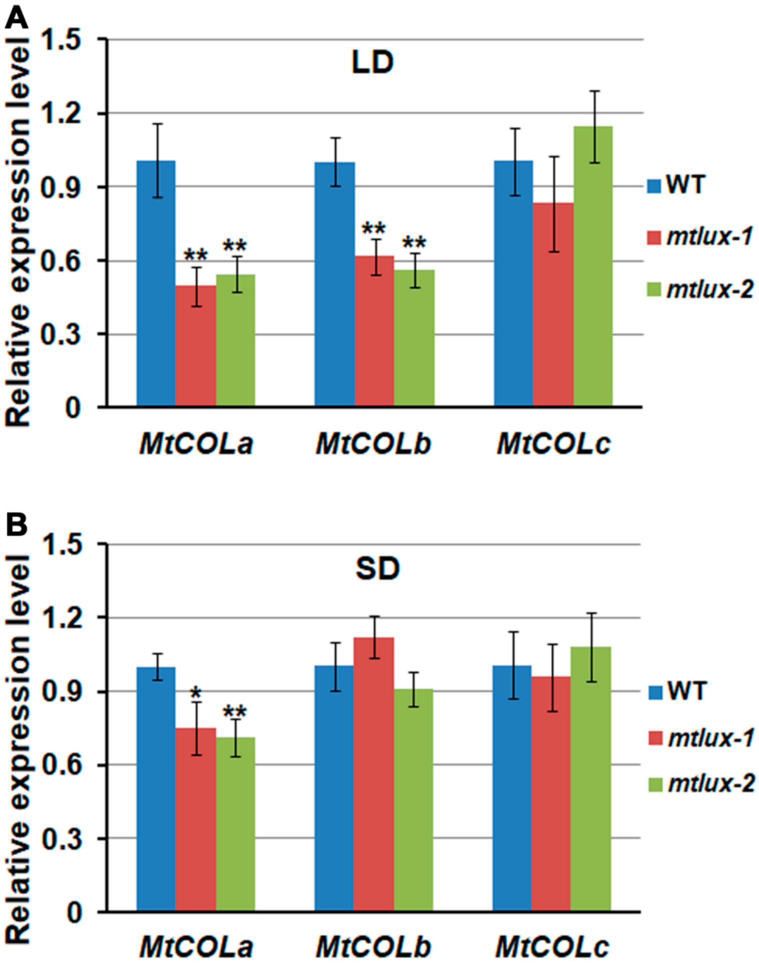

Figure S5 | Relative gene expression of three *Medicago COL* genes in long day (A) and short day (B) conditions in WT and *mtlux* mutant plants. Values shown as means ± SD of three biological replicates. *, **: means differ significantly ($P < 0.05$, $P < 0.01$).

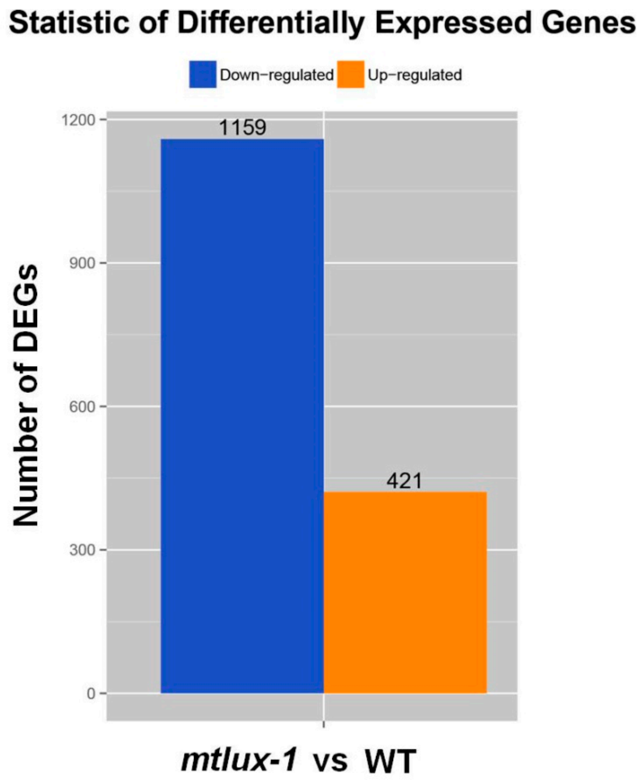

Figure S6 | Differentially expressed genes (DEGs) between *mtlux-1* and wild type.

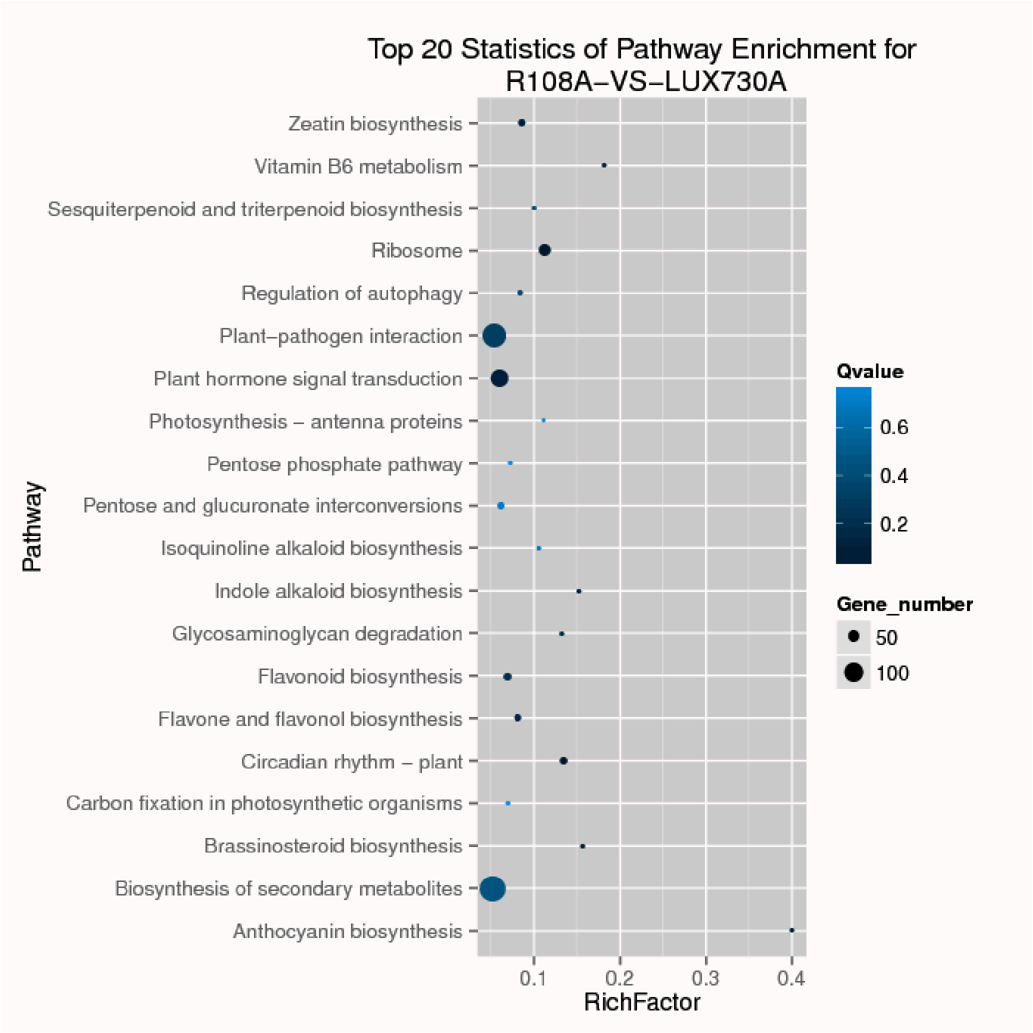

Figure S7 | Functional assignment of the differentially expressed genes (DEGs) by Gene Ontology (GO) analysis of top20 pathways. R108A as the wild type and LUX730A as the *mtlux-1* mutant. Scale represents *q* value.

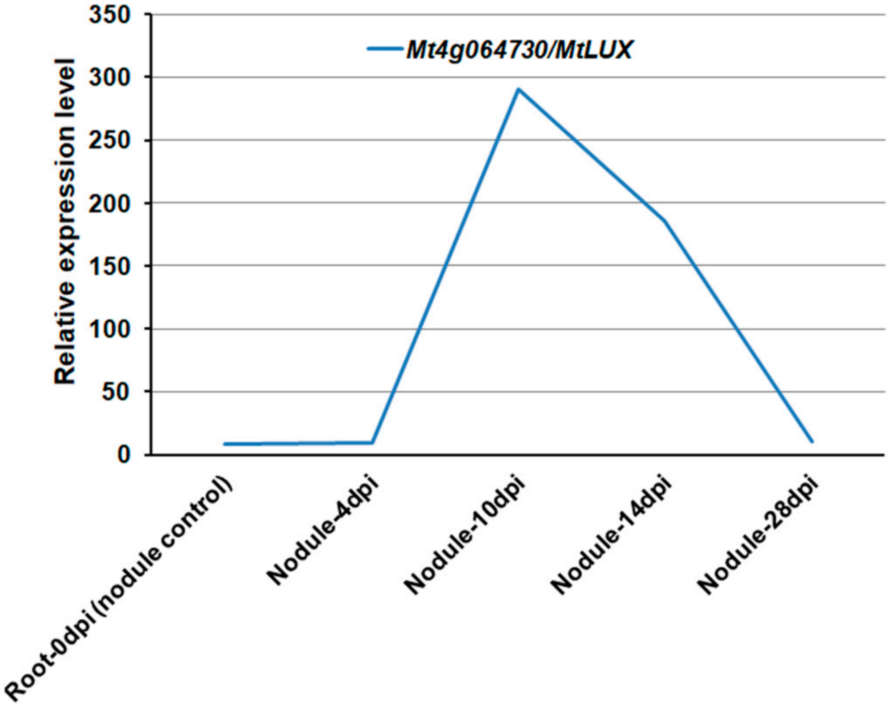

Figure S8 | Transcriptional profile of *MtLUX* in nodule. The transcriptional profile of *MtLUX* derived from the *M. truncatula* Gene Expression Atlas (https://mtgea.noble.org/v2/). The microarray probe set used is Mtr.32211.1.S1_at.
